# Circulating Cit-H3 and MPO-DNA complexes reveal divergent neutrophil-associated immune profiles in multiple myeloma

**DOI:** 10.3389/fimmu.2026.1960912

**Published:** 2026-09-25

**Authors:** Jingdi Liu, Li Cai, Jiao Qu, Chunyan Sun, Qiubai Li, Lei Chen

**Affiliations:** 1 Department of Hematology, Union Hospital, Tongji Medical College, Huazhong University of Science and Technology, Wuhan, China; 2 Department of Rheumatology and Immunology, Union Hospital, Tongji Medical College, Huazhong University of Science and Technology, Wuhan, China

**Keywords:** biomarker, citrullinated histone H3, MPO-DNA complexes, multiple myeloma, neutrophil extracellular traps, tumor microenvironment

## Abstract

**Introduction:**

Neutrophils are increasingly recognized as dynamic regulators of tumor inflammation and immune remodeling. Neutrophil extracellular trap (NET)-related biomarkers have been implicated in cancer progression, but their clinical and immunological interpretation remains challenging because different circulating NET components may not represent equivalent biological processes. In multiple myeloma, a malignancy shaped by complex interactions among clonal plasma cells, hematopoiesis, and the bone marrow immune microenvironment, the relevance of distinct circulating NET-related markers remains unclear.

**Methods:**

We measured serum citrullinated histone H3 (Cit-H3) and myeloperoxidase-DNA complexes (MPO-DNA) in 171 patients with multiple myeloma and 15 healthy controls. Patients were categorized by disease status at sampling as newly diagnosed multiple myeloma, remission, or relapsed/refractory multiple myeloma. Associations with disease status, inflammatory parameters, tumor burden, hematologic indices, R-ISS and R2-ISS stage, high-risk cytogenetic abnormalities, and exploratory survival outcomes were analyzed.

**Results:**

Serum Cit-H3 levels were significantly higher in patients with multiple myeloma than in healthy controls and were highest in relapsed/refractory disease. Cit-H3 was associated with lower lymphocyte counts and higher neutrophil-to-lymphocyte ratios. In contrast, serum MPO-DNA complexes were not significantly increased in the overall myeloma cohort compared with controls and were lower in relapsed/refractory disease than in newly diagnosed or remission states. In exploratory correlation analyses, higher MPO-DNA levels were associated with higher hemoglobin and albumin levels, lower bone marrow plasma cell burden, and lower β2-microglobulin. Cit-H3 and MPO-DNA levels were not significantly correlated, and neither marker was significantly associated with R-ISS or R2-ISS stage, del(17p), t(4;14), or 1q21 amplification. Exploratory survival analyses showed no statistically significant associations with progression-free or overall survival.

**Discussion:**

Circulating Cit-H3 and MPO-DNA complexes are not interchangeable NET-related biomarkers in multiple myeloma and do not simply reflect conventional clinical or cytogenetic risk stratification. Rather than representing a uniform NET signal, these markers may capture distinct aspects of neutrophil-related immune dysregulation, systemic inflammation, and hematopoietic status, highlighting the need for prospective validation.

## 1 Introduction

Multiple myeloma (MM) is an incurable hematologic malignancy characterized by clonal expansion of plasma cells within the bone marrow and profound remodeling of the local and systemic immune environment. Although novel therapeutics have substantially extended survival, relapse remains a major challenge, highlighting the biological complexity of myeloma and the importance of immune and microenvironmental factors in disease evolution (1, 2). Current staging and risk stratification predominantly focus on intrinsic features including cytogenetic profiles of malignant plasma cells (3, 4). However, more evidence indicates that non-malignant components of the tumor microenvironment (TME) also contribute to disease progression, immune escape, and treatment resistance (5, 6).

The tumor microenvironment in multiple myeloma involves complex interactions among malignant plasma cells, stromal cells, T cells, natural killer cells, monocytes, macrophages, and neutrophils (7). Among these components, neutrophils have traditionally received less attention. However, neutrophils are now recognized as highly heterogeneous and plastic cells in cancer rather than a uniform effector population. Depending on disease status, systemic inflammatory signals, tumor burden, and treatment exposure, neutrophils may acquire diverse functional states associated with immune suppression, tissue remodeling, thrombosis, angiogenesis, or antitumor activity (8). Therefore, neutrophil-related biomarkers may provide additional insight into the systemic immune state of patients with cancer.

A hallmark of neutrophil activation is the formation of neutrophil extracellular traps (NETs). NETs are extracellular structures composed of chromatin, histones, and neutrophil granule proteins. In cancer, NETs have been implicated in tumor inflammation, immune evasion, thrombosis, and resistance to therapy. Nevertheless, translating NET biology into clinically interpretable biomarkers remains difficult. Circulating NET markers are heterogeneous and may differ in their cellular origin, release mechanisms, extracellular stability, degradation, and clearance (9–11). As a result, individual NET biomarkers may not be interchangeable, particularly in complex clinical settings such as hematologic malignancies.

Although citrullinated histone H3 (Cit-H3) and myeloperoxidase-DNA (MPO-DNA) complexes are both widely used circulating NET-related markers, they reflect distinct molecular facets of NET biology. Cit-H3 is associated with PAD4-mediated histone citrullination and chromatin decondensation, whereas MPO-DNA complexes reflect the association between neutrophil granule proteins and extracellular DNA (12, 13). Despite intense interest in NETs in cancer immunity, the clinical and immunological relevance of distinct circulating NET-related biomarkers in multiple myeloma remains undefined. It is unclear whether Cit-H3 and MPO-DNA complexes show similar patterns across disease states or whether they reflect different immune-inflammatory or hematopoietic features. Addressing this question may help clarify how circulating NET-related biomarkers should be interpreted in myeloma and may provide a basis for future studies integrating serum biomarkers with bone marrow immune profiling.

In this study, we measured serum Cit-H3 and MPO-DNA levels in patients with newly diagnosed, remission, and relapsed/refractory MM states. We analyzed their associations with disease status, immune-inflammatory parameters, hematologic indices, tumor burden, high-risk cytogenetic abnormalities, and exploratory survival outcomes. We aimed to determine whether these two circulating NET-related markers represent overlapping or divergent neutrophil-associated immune profiles in multiple myeloma.

## 2 Materials and methods

### 2.1 Study population and sample collection

This was a single-center, retrospective observational study conducted at Union Hospital. Between 2019 and 2023, a total of 186 participants were enrolled, comprising 171 patients with multiple myeloma and 15 healthy controls. The MM cohort was stratified into three subgroups based on disease status at the time of blood sampling: newly diagnosed MM (NDMM, n = 82), relapsed and/or refractory MM (RRMM, n = 39), and patients in very good partial response or complete remission (Remission, n = 50).

NDMM samples were collected at diagnosis before the initiation of systemic anti-myeloma therapy. Remission samples were collected during routine follow-up from patients who had achieved very good partial response or complete response according to IMWG response criteria. RRMM was defined as documented relapse or disease progression after prior anti-myeloma therapy based on IMWG criteria and treating physician assessment, and samples were obtained at the time of clinical evaluation for relapse or progression (14, 15). Available medical records were reviewed for intercurrent inflammatory conditions and recent therapies that could potentially influence neutrophil-related biomarkers. Given the retrospective real-world nature of the cohort, these factors could not be fully standardized or used as strict exclusion criteria; therefore, they were considered in the interpretation of the results, and sensitivity analyses were performed where appropriate.

The retrospective study was approved by the Institutional Ethics Committee of Union Hospital, Tongji Medical College, Huazhong University of Science and Technology (approval No [2026]. LunShenZi (0200)) and the requirement for informed consent was waived.

Peripheral venous blood samples had been collected from patients and healthy controls as part of routine clinical assessment or during diagnostic workup. Blood was allowed to clot at room temperature for 30 minutes, and serum was separated by centrifugation at 3000 g for 10 minutes at room temperature. Serum aliquots were stored at −80 °C until analysis and were thawed only once before the ELISA testing.

### 2.2 Quantification of NET-related markers by ELISA

#### 2.2.1 Serum MPO-DNA complex detection

Serum MPO–DNA complexes were measured by a capture ELISA adapted from previously published methods (16). Briefly, 96-well microtiter plates were coated with anti-MPO monoclonal antibody (ABD Serotec, 0400-0002) at 5 μg/mL and incubated overnight at 4 °C. Plates were blocked with 1% bovine serum albumin (BSA). Samples were then added and incubated, followed by detection with a peroxidase-conjugated anti-DNA antibody (Cell Death Detection ELISA kit component 2, Roche, 1177445001). After washing (three times with PBS), peroxidase substrate was added and plates were incubated at 37 °C for 40 minutes. Optical density was read at 405 nm on a microplate reader (Tecan Infinite F50). MPO–DNA results were expressed as OD405 arbitrary units. The assay was performed with appropriate controls and in duplicate.

#### 2.2.2 Serum citrullinated histone H3 detection

Cit-H3 was quantified in serum using the Citrullinated Histone H3 (clone 11D3) ELISA Kit (Cayman, 501620) according to the manufacturer’s instructions. Absorbance was read at 450 nm.

All samples were measured in duplicate, and the mean value was used for analysis. Samples from different disease groups were distributed across plates to minimize batch effects.

### 2.3 Clinical data collection

Clinical and laboratory parameters were extracted from electronic medical records, including: Revised International Staging System (R-ISS) classification, bone marrow plasma cell percentage (morphology and flow cytometry), complete blood count (hemoglobin, white blood cell count, neutrophil count, lymphocyte count, platelet count), C-reactive protein (CRP), hepatic function markers (albumin, globulin), renal function markers (estimated glomerular filtration rate), β2 microglobulin (β2-MG), corrected calcium and lactate dehydrogenase (LDH). The neutrophil-to-lymphocyte ratio (NLR) was calculated as the absolute neutrophil count divided by the absolute lymphocyte count.

High-risk cytogenetic abnormalities (del(17p), t(4;14), and 1q21 amplification) were assessed by fluorescence *in situ* hybridization (FISH) performed on bone marrow samples at diagnosis, according to routine clinical procedures. R2-ISS stage was calculated according to the Second Revision of the International Staging System when the required variables were available.

### 2.4 Follow-up and study endpoints

Patients were followed by outpatient visits and review of electronic medical records. Progression-free survival (PFS) was defined as time from diagnosis to disease progression or death from any cause. Overall survival (OS) was defined as time from diagnosis to death from any cause. OS data were considered evaluable only when vital status could be reliably confirmed. Patients without events were censored at the date of last follow-up.

### 2.5 Definition of NET profile groups for exploratory survival analysis

For exploratory survival analyses, NDMM patients with available follow-up data were categorized based on baseline (at diagnosis) serum levels of MPO-DNA and Cit-H3, using exploratory thresholds derived from healthy control values and the NDMM cohort distribution.

#### 2.5.1 MPO-DNA stratification

We used the mean MPO-DNA level of the 15 healthy controls (mean = 1.024) as a reference for physiological baseline levels. NDMM patients were classified into two groups: high MPO-DNA: baseline serum level ≥ healthy control mean; low MPO-DNA: baseline serum level < healthy control mean.

#### 2.5.2 Cit-H3 stratification

In the absence of validated clinical cutoffs, we stratified patients using the tertile distribution of baseline Cit-H3 levels within the NDMM cohort. Specifically: high Cit-H3: upper tertile (≥0.505, representing the top 33% of patients); low Cit-H3: lower two tertiles (<0.505, representing the bottom 67% of patients).

To explore the potential synergistic prognostic value of the two NET components, patients were further classified into three composite categories:

High MPO-DNA and low Cit-H3 group;

Other combinations group (high MPO-DNA/high Cit-H3 or low MPO-DNA/low Cit-H3);

Low MPO-DNA and high Cit-H3 group.

This composite grouping was derived from our cohort data as an exploratory approach to account for the opposing trends of Cit-H3 and MPO-DNA (see Results section 3.4).

### 2.6 Statistical analysis

Statistical analyses were performed using GraphPad Prism 9 and R software (version 4.3.1). Continuous variables were summarized as median (range) or median (interquartile range), as appropriate. Comparisons among multiple groups were performed using the Kruskal-Wallis test, followed by *post hoc* pairwise comparisons with Bonferroni correction when appropriate. Comparisons between two groups were conducted using the Mann-Whitney U test. Categorical variables were compared using the chi-square test or Fisher’s exact test.

Correlations between NET-related biomarkers and clinical variables were evaluated using Spearman’s rank correlation analysis. To account for multiple exploratory comparisons, nominal p values were adjusted using the Benjamini-Hochberg false discovery rate method. FDR-adjusted q values are reported alongside nominal p values. Associations between NET-related biomarkers and R-ISS or R2-ISS stages in NDMM patients were assessed using Spearman rank correlation analysis. To assess whether the association between MPO-DNA and neutrophil count was independent of disease status, an exploratory multivariable linear regression model was fitted in the entire MM cohort. MPO-DNA was entered as the dependent variable, and neutrophil count and disease status were included as predictors. Disease status was treated as a categorical variable and entered using two dummy variables, with NDMM as the reference category. Regression coefficients are reported as unstandardized B values with 95% confidence intervals and standardized β coefficients. Kaplan–Meier methods were used to estimate PFS and OS, and survival curves were compared using the log-rank test. Univariable Cox proportional hazards regression models were used for exploratory survival analyses. All tests were two-sided, and p < 0.05 were considered statistically significant.

## 3 Results

### 3.1 Baseline characteristics

The baseline characteristics of the study cohort are summarized in **Table 1**. Age and sex distribution were comparable among NDMM, remission, and RRMM groups. As expected, disease-related parameters, including bone marrow plasma cell burden, hemoglobin, platelet count, involved/uninvolved free light chain ratio, β2-microglobulin, albumin, corrected calcium, and eGFR, differed across disease states. Several additional laboratory parameters, including lymphocyte count, CRP, and LDH, also differed among groups and were considered descriptive baseline characteristics.

**Table 1:** Clinical characteristics of patients with multiple myeloma according to disease status at sampling.

Variable	NDMM (n=82)	Remission (n=50)	RRMM (n=39)	p value
Demographic characteristics				
Age, years, median (range)	60 (38-73)	59 (43-75)	61 (44-77)	0.384
Male sex, n (%)	44 (53.7)	25 (50.0)	24 (61.5)	0.427
Disease-related characteristics				
R-ISS stage at diagnosis, n (%) [available n = 75]				
I	15 (20.0)	–	–	–
II	48 (64.0)	–	–	–
III	12 (16.0)	–	–	–
Tumor burden at sampling				
Bone marrow plasma cells by morphology, %, median (range)	36.5 (11-75)	0 (0-0)	16.0 (1.0-60.0)	<0.001
Bone marrow plasma cells by flow cytometry, %, median (range)	20.79 (8.9-76.7)	0 (0-0)	5.92 (0.49-69.37)	<0.001
High-risk cytogenetic abnormalities in NDMM at diagnosis				
del(17p), n/N (%)	5/77 (6.5)	–	–	–
t(4;14), n/N (%)	16/74 (21.6)	–	–	–
1q21 amplification, n/N (%)	29/77 (37.7)	–	–	–
Laboratory parameters at sampling, median (range)				
WBC, x10^9^/L	4.93 (1.78-11.1)	5.26 (2.06-15.49)	4.80 (0.85-21.47)	0.503
Neutrophils, x10^9^/L	2.97 (1.3-7.56)	3.02 (0.63-13.64)	2.85 (0.38-13.7)	0.893
Lymphocytes, x10^9^/L	1.57 (0.3-4.99)	1.21 (0.56-2.57)	1.37 (0.37-3.96)	0.021
NLR	2.04 (0.81-7.26)	2.25 (0.72-10.03)	2.02 (0.49-13.03)	0.390
Hemoglobin, g/L	95 (48-155)	124 (79-144)	101 (52-169)	<0.001
Platelets, x10^9^/L	192 (43-525)	186 (42-415)	134 (13-247)	<0.001
Involved/uninvolved free light chain ratio	53.49 (0.93-11849.7)	1.15 (0.69-1.93)	16.46 (0.60-1460.18)	<0.001
β2-microglobulin, mg/L	4.0 (1.8-59.5)	2.7 (1.8-6.1)	5.0 (1.9-41.9)	<0.001
CRP, mg/L	3.14 (0.11-82.1)	3.02 (0.27-5.45)	4.54 (0.37-99.17)	0.009
CRP >10 mg/L, n/N (%)	8/82 (9.8)	0/50 (0)	5/39 (12.8)	0.046
Albumin, g/L	36.7 (19.8-46.9)	40.3 (32.8-48.8)	38.1 (23.9-47.2)	0.001
LDH, U/L	175 (88-698)	205 (112-376)	196 (83-660)	0.010
Corrected calcium, mmol/L	2.34 (1.93-3.34)	2.19 (1.96-2.48)	2.28 (1.98-3.52)	<0.001
eGFR, mL/min/1.73m^2^	78.44 (3.36-113.98)	95.23 (55.84-116.05)	77.29 (3.50-115.76)	0.004

Data are presented as median (range) or n/N (%), as appropriate. P values indicate comparisons among NDMM, remission, and RRMM groups. Continuous variables were compared using the Kruskal–Wallis test, and categorical variables were compared using the chi-square test or Fisher’s exact test, as appropriate. High-risk cytogenetic abnormalities were analyzed only in the NDMM cohort using FISH results obtained at diagnosis. Elevated CRP was defined as CRP >10 mg/L. NDMM, newly diagnosed multiple myeloma; RRMM, relapsed/refractory multiple myeloma; R-ISS, Revised International Staging System; WBC, white blood cell count; NLR, neutrophil-to-lymphocyte ratio; CRP, C-reactive protein; LDH, lactate dehydrogenase; eGFR, estimated glomerular filtration rate.

### 3.2 Disease stage-specific dynamics of circulating NET-related biomarkers

Serum Cit-H3 levels were significantly elevated in MM patients compared with healthy controls (overall MM vs Control, p <0.001). Median (IQR) serum Cit-H3 levels were 0.173 (0.167, 0.186) in controls, 0.371 (0.230, 0.733) in NDMM, 0.476 (0.274, 0.695) in remission, and 0.882 (0.551, 1.192) in RRMM. Within the MM cohort, Cit-H3 was highest in the RRMM group and was significantly greater than in both the NDMM and the Remission groups (RRMM vs NDMM and RRMM vs Remission, both p <0.001). There was no significant difference between NDMM and Remission (**Figures 1A, B**).

**Figure 1:**
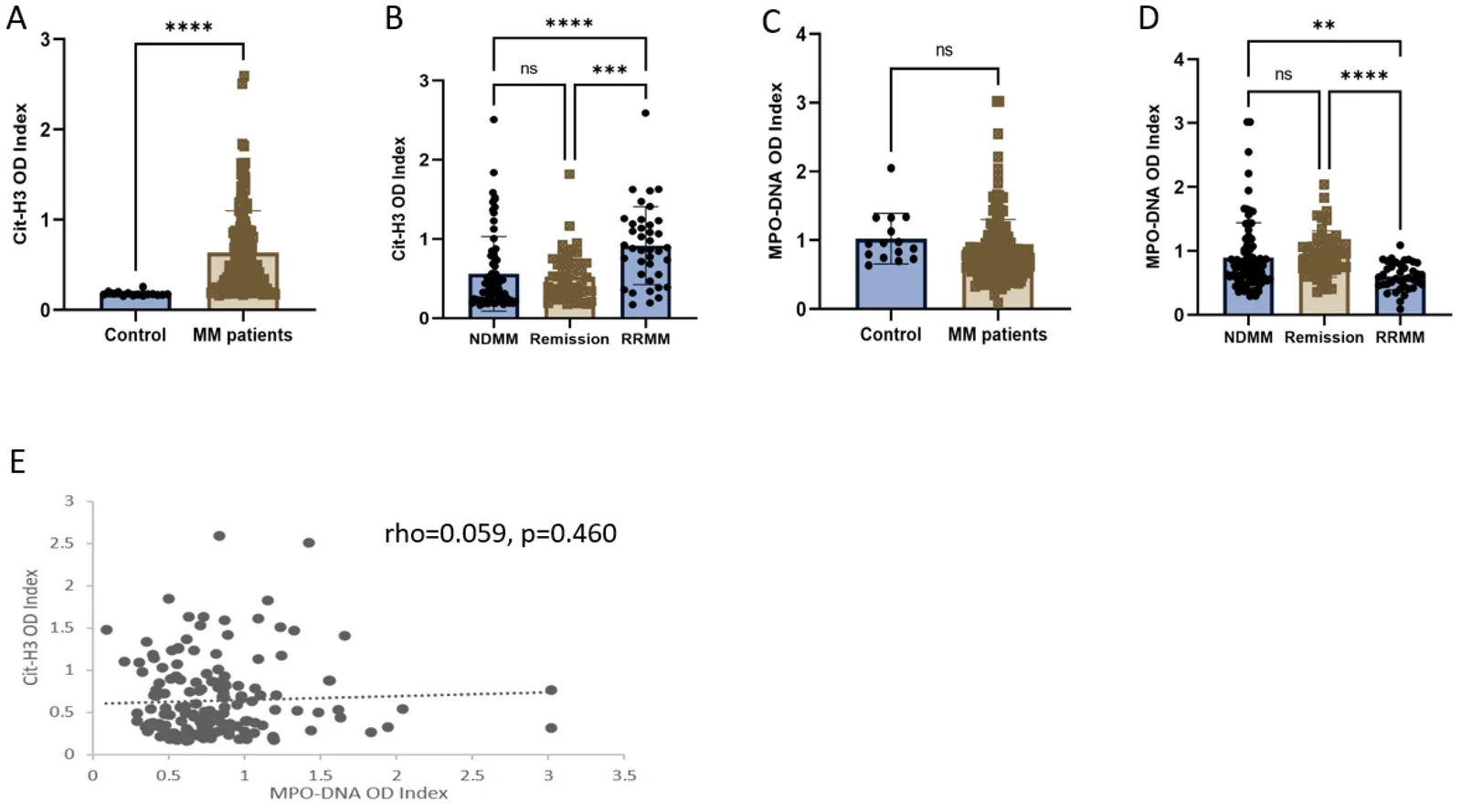
Serum Cit-H3 and MPO-DNA levels and their relationship across study groups. **(A, B)** Serum citrullinated histone H3 (Cit-H3) levels in MM patients stratified by disease status and compared with healthy controls. **(C, D)** Serum MPO-DNA complex levels in MM patients stratified by disease status and compared with healthy controls. **(E)** Scatter plot demonstrating the absence of significant correlation between serum Cit-H3 and MPO-DNA levels across the MM cohort. Correlation was assessed using Spearman rank analysis. Statistical significance is indicated as: ns, not significant; *p <0.05; **p <0.01; ***p <0.001; ****p <0.0001.

By contrast, serum MPO–DNA levels did not differ between the pooled MM population and healthy controls. Median (IQR) serum MPO-DNA levels were 0.949 (0.745, 1.329) in controls, 0.738 (0.559, 1.018) in NDMM, 0.884 (0.709, 1.126) in remission, and 0.579 (0.460, 0.787) in RRMM. The pattern among MM subgroups was the inverse of that observed for Cit-H3: MPO–DNA was significantly lower in the RRMM group than in NDMM and Remission (RRMM vs NDMM, p = 0.007; RRMM vs Remission, p <0.001). MPO–DNA tended to be higher in Remission than in NDMM, but this comparison did not reach statistical significance (Remission vs NDMM, p = 0.059) (**Figures 1C, D**).

Because circulating NET-related markers may be influenced by acute inflammatory conditions, we further performed a sensitivity analysis excluding patients with CRP levels >10 mg/L. After this exclusion, Cit-H3 remained significantly elevated in RRMM compared with both NDMM (p = 0.004) and remission (p = 0.019). MPO-DNA levels also remained significantly lower in RRMM than in remission patients (p = 0.003), while the comparison between RRMM and NDMM showed a non-significant trend in the same direction (p = 0.076). These results indicate that the main disease-state-associated differences, particularly the elevation of Cit-H3 in RRMM, persisted after removal of patients with markedly increased CRP.

### 3.3 Cit-H3 and MPO-DNA complexes represent non-overlapping circulating NET profiles

To investigate the relationship between these two distinct NET-related components, we performed a correlation analysis across the entire MM patient cohort. Notably, serum Cit-H3 and MPO-DNA levels showed no significant correlation (Spearman rho = 0.059, p = 0.460; **Figure 1E**). This lack of association suggests that Cit-H3 and MPO-DNA may reflect distinct aspects of neutrophil activation and NETosis dynamics in MM, potentially providing complementary rather than redundant information.

### 3.4 Distinct immune-inflammatory and hematologic associations of Cit-H3 and MPO-DNA complexes

Spearman correlation analyses revealed that serum Cit-H3 and MPO-DNA exhibit distinct clinical profiles (**Table 2**). These correlation analyses were exploratory and were not adjusted for disease status or treatment exposure. Cit-H3 showed associations mainly with inflammatory and immune parameters. It showed a negative correlation with absolute lymphocyte count and a positive correlation with the neutrophil-to-lymphocyte ratio (NLR), aligning with a systemic inflammatory phenotype. The association between Cit-H3 and NLR remained significant after FDR correction.

**Table 2:** Spearman correlation of serum Cit-H3 and MPO-DNA levels with clinical parameters in patients with multiple myeloma.

Clinical parameter	Cit-H3, rho	Cit-H3, p	Cit-H3, q	MPO-DNA, rho	MPO-DNA, p	MPO-DNA, q
Age	0.088	0.266	0.703	0.005	0.945	0.945
Bone marrow plasma cells by morphology, %	0.182	0.091	0.364	-0.370	**<0.001**	**0.004**
Bone marrow plasma cells by flow cytometry, %	0.113	0.318	0.703	-0.332	**0.002**	**0.006**
WBC, x10^9^/L	-0.065	0.418	0.703	0.296	**<0.001**	**0.004**
Neutrophils, x10^9^/L	0.052	0.522	0.703	0.310	**<0.001**	**0.004**
Lymphocytes, x10^9^/L	-0.307	**<0.001**	**0.016**	-0.023	0.789	0.842
Hemoglobin, g/L	0.053	0.514	0.703	0.286	**<0.001**	**0.004**
Platelets, x10^9^/L	-0.159	0.069	0.364	0.154	0.071	0.118
NLR	0.256	**0.003**	**0.024**	0.228	**0.007**	**0.019**
CRP	0.035	0.728	0.843	0.033	0.738	0.842
Albumin, g/L	-0.083	0.305	0.703	0.187	**0.017**	**0.039**
β2-microglobulin, mg/L	-0.054	0.527	0.703	-0.190	**0.022**	**0.044**
Involved/uninvolved free light chain ratio	0.063	0.446	0.703	-0.073	0.399	0.491
LDH, U/L	0.030	0.738	0.843	0.098	0.257	0.343
Corrected calcium, mmol/L	-0.008	0.922	0.946	-0.141	0.074	0.118
eGFR, mL/min/1.73m^2^	-0.008	0.946	0.946	0.142	0.208	0.303

Correlations were assessed using Spearman rank correlation analysis. False discovery rate correction was performed separately for Cit-H3 and MPO-DNA correlation analyses using the Benjamini–Hochberg method. P values reported as p < 0.001 were conservatively entered as 0.001 for FDR correction. Both nominal p values and FDR-adjusted q values are shown. Statistically significant correlations (p <0.05) are highlighted in bold.

Conversely, MPO-DNA correlated broadly with markers of hematologic function and disease burden. Elevated MPO-DNA positively correlated with white blood cell count, neutrophil count, hemoglobin, and albumin, while negatively correlating with bone marrow plasma cell percentage and β2-microglobulin. This pattern suggests an association with more favorable hematologic status and lower tumor burden. Although MPO-DNA also positively correlated with NLR, this likely reflects its mathematical dependence on the absolute neutrophil count rather than true systemic inflammation. MPO-DNA was positively associated with neutrophil count and hemoglobin and inversely associated with bone marrow plasma cell burden. These associations remained significant after FDR correction.

We next stratified patients using predefined clinically relevant cutoffs. MPO-DNA levels were significantly higher in non-anemic patients (hemoglobin ≥90 g/L; p = 0.001, **Figure 2A**) and those with normal albumin (≥35 g/L; p = 0.033, **Figure 2B**). Meanwhile, Cit-H3 levels were significantly elevated in patients with lymphocytopenia (lymphocyte count <1.0 × 10^9^/L; p = 0.002, **Figure 2C**) and those with an NLR ≥3 (p = 0.003, **Figure 2D**).

**Figure 2:**
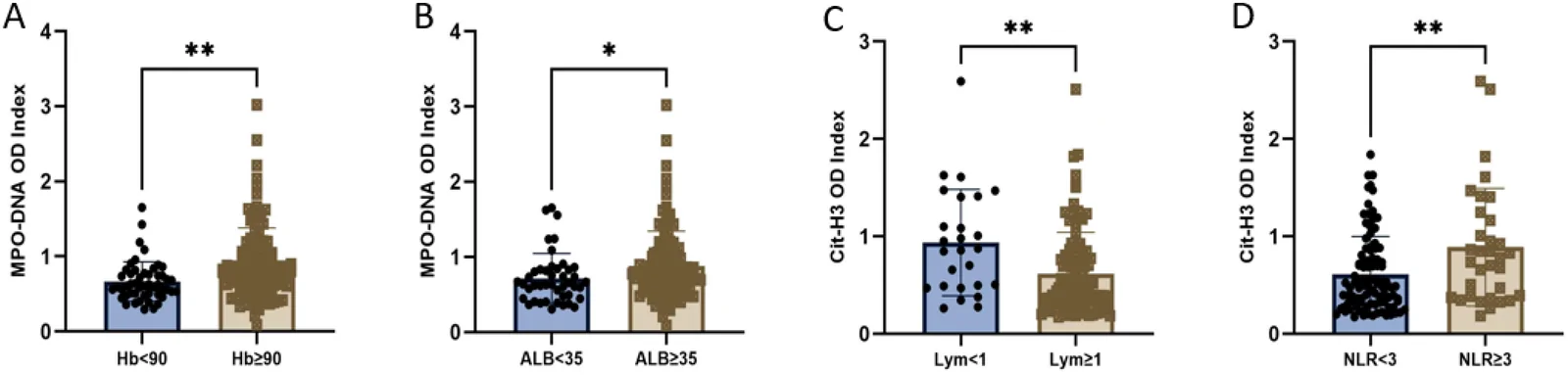
Stratification analysis of serum Cit-H3 and MPO-DNA by predefined clinical cutoffs. **(A)** Serum MPO-DNA levels are significantly higher in non-anemic patients (hemoglobin ≥90 g/L) compared to anemic patients (hemoglobin <90 g/L). **(B)** Serum MPO-DNA levels are significantly higher in patients with normal serum albumin (≥35 g/L) compared to patients with hypoalbuminemia (<35 g/L). **(C)** Serum Cit-H3 levels are significantly elevated in patients with lymphocytopenia (lymphocyte count <1.0×10^9^/L) compared to patients with normal lymphocyte counts (≥1.0×10^9^/L). **(D)** Serum Cit-H3 levels are significantly elevated in patients with elevated systemic inflammation (NLR ≥3) compared to patients with lower NLR (<3). Comparisons between groups were performed using the Mann–Whitney U test.

Overall, these analyses indicate that Cit-H3 is primarily linked to an inflammatory phenotype, whereas MPO-DNA is associated with lower tumor burden and more favorable hematologic and nutritional parameters.

To assess whether the observed association between MPO-DNA and neutrophil count was independent of disease status, we performed a multivariable linear regression analysis in the entire MM cohort. Serum MPO-DNA was used as the dependent variable, with neutrophil count and disease status included as predictors. Disease status was entered as a categorical variable using two dummy variables, with NDMM as the reference category. The overall model was statistically significant (p = 0.001). However, neutrophil count was not significantly associated with MPO-DNA levels after adjustment for disease status (B = 0.022, 95% CI -0.010 to 0.053, standardized β = 0.098, p = 0.184). The remission group did not differ significantly from NDMM (B = 0.057, 95% CI -0.099 to 0.213, standardized β = 0.057, p = 0.472), whereas the RRMM group had significantly lower MPO-DNA levels than the NDMM group (B = -0.292, 95% CI -0.461 to -0.122, standardized β = -0.267, p = 0.001). These findings suggest that the unadjusted association between MPO-DNA and neutrophil count may be partly attributable to disease-state heterogeneity, while the lower MPO-DNA levels observed in RRMM persisted after adjustment for neutrophil count.

### 3.5 Circulating NET-related markers are not associated with clinical risk stratification or high-risk cytogenetic abnormalities

We next examined whether baseline NET-related markers in NDMM were associated with established myeloma risk features, including R-ISS stage, R2-ISS stage, and common high-risk cytogenetic abnormalities detected by FISH. No significant correlations were observed between R-ISS stage and Cit-H3 (p = 0.230) or MPO-DNA (p = 0.486), nor between R2-ISS stage and Cit-H3 (p = 0.429) or MPO-DNA (p = 0.663).

We further analyzed high-risk cytogenetic abnormalities, including del(17p), t(4;14), and 1q21 amplification. There were no significant differences in serum Cit-H3 between patients with or without del(17p) (p = 0.172), t(4;14) (p = 0.078), or 1q21 amplification (p = 0.220). Similarly, serum MPO-DNA did not differ significantly according to del(17p) (p = 0.695), t(4;14) (p = 0.414), or 1q21 amplification (p = 0.800).

Overall, no significant associations were observed between circulating NET-related markers and established myeloma risk stratification systems or common high-risk cytogenetic abnormalities in this cohort. These findings suggest that Cit-H3 and MPO-DNA may capture systemic immune-inflammatory or hematopoietic features not directly reflected by conventional plasma cell-intrinsic risk markers, although this interpretation requires validation in larger NDMM cohorts.

### 3.6 Exploratory outcome analysis of combined NET immune profiles

Survival data were available for a subset of the NDMM cohort. Patients were diagnosed between January 2019 and December 2021. Follow-up data were obtained through retrospective chart review, including scheduled outpatient visit records, hospitalization records, and death registry. The data cutoff date for survival analysis was December 31, 2025. Among the 82 NDMM patients, complete follow-up data were available for 55 patients (67.1%) for PFS analysis and 44 patients (53.7%) for OS analysis. For those with evaluable data, the median follow-up duration was 55 months (range: 48–72 months) for PFS and 55 months (range: 48–72 months) for OS.

In univariable Cox proportional-hazards regression models, neither baseline Cit-H3 nor MPO-DNA, analyzed as continuous variables, was a significant predictor of PFS or OS (all p > 0.05). R-ISS stage and age also did not reach statistical significance in this subset, likely reflecting the limited number of events and sample size.

As an exploratory, hypothesis-driven analysis, we categorized patients at diagnosis into three groups according to the combined baseline NET profile: high MPO-DNA/low Cit-H3 group, other combinations group, and low MPO-DNA/high Cit-H3 group.

For PFS, the high MPO-DNA/low Cit-H3 group (n = 5) showed the most favorable survival pattern; only one PFS event occurred in this group and the median PFS was not reached (not estimable). In contrast, the median PFS was 31 months in other combinations group (n = 33; 26 events) and 26 months in the low MPO-DNA/high Cit-H3 group (n = 17; 13 events). The overall comparison among the three groups did not reach statistical significance (log-rank p = 0.141; **Figure 3A**). For OS, no deaths occurred in the high MPO-DNA/low Cit-H3 group (n = 4; 0 events), and the median OS was not reached in any group (other combinations group: n = 27, 10 events; low MPO-DNA/high Cit-H3 group: n = 13, 4 events). Although the Kaplan–Meier curves suggested a trend toward better OS in the high MPO-DNA/low Cit-H3 group, the difference was not statistically significant (log-rank p = 0.603; **Figure 3B**).

**Figure 3:**
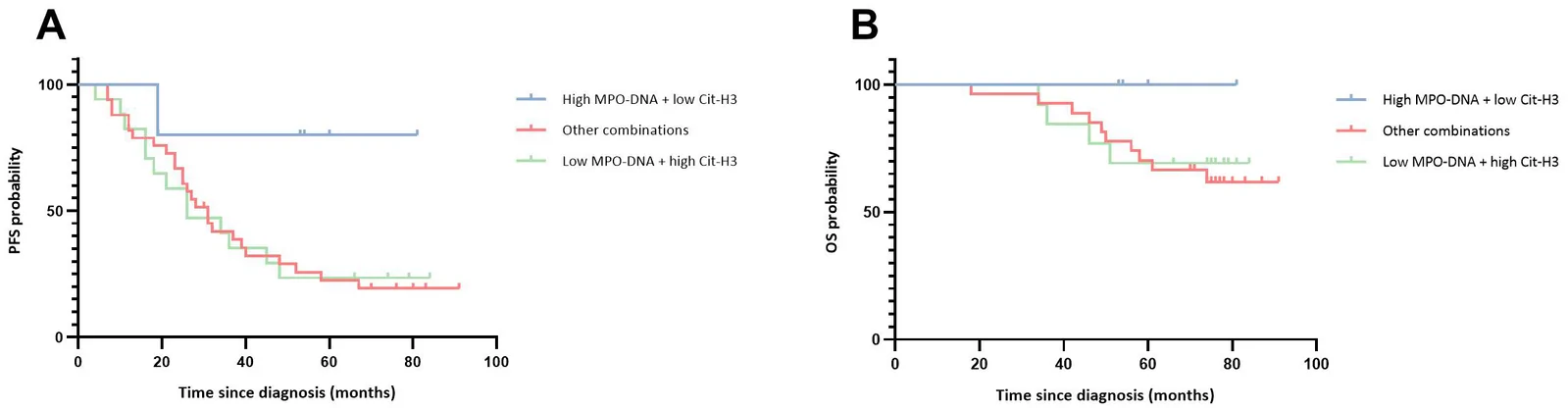
Exploratory Kaplan-Meier survival analysis of NDMM patients stratified by combined NETs profile. **(A)** Progression-free survival (PFS) for high MPO-DNA/low Cit-H3 group, other combinations group, and low MPO-DNA/high Cit-H3 group. **(B)** Overall survival (OS) curves for the same groups.

Given the very small size of the high MPO-DNA/low Cit-H3 group and substantial censoring, these estimates should be interpreted cautiously. Overall, these survival analyses are exploratory and hypothesis-generating.

## 4 Discussion

In this study, we found that two commonly used circulating NET-related biomarkers, Cit-H3 and MPO-DNA complexes, showed divergent clinical and immune-associated profiles in multiple myeloma. Cit-H3 was most elevated in relapsed/refractory disease and was associated with lymphopenia and elevated NLR. Conversely, serum MPO-DNA complexes were lower in relapsed/refractory disease and were associated with more preserved hematologic parameters and lower tumor burden. These findings suggest that circulating NET-related biomarkers should not be interpreted as a uniform NET signal in multiple myeloma.

The elevation of Cit-H3 in relapsed/refractory multiple myeloma may reflect an intensified neutrophil-associated inflammatory state in advanced or treatment resistant disease. Its association with lower lymphocyte counts and higher NLR further suggests a link between Cit-H3 and systemic inflammatory imbalance. Notably, because our study measured circulating serum markers, these findings should not be interpreted as direct evidence of increased NET deposition within the bone marrow.

The association of higher MPO-DNA levels with lower tumor burden and more favorable hematologic parameters was unexpected if circulating MPO-DNA is viewed solely as a marker of pathogenic NET formation. One possible interpretation is that circulating MPO-DNA levels may be influenced by neutrophil availability and preserved hematopoietic function. However, although MPO-DNA was positively correlated with neutrophil count in the unadjusted analysis, neutrophil count was no longer statistically associated with MPO-DNA after adjustment for disease status. This finding suggests that disease-state heterogeneity may partly account for the observed unadjusted association. In patients with extensive marrow involvement or relapsed/refractory disease, impaired hematopoiesis, altered neutrophil fitness, treatment exposure, or differences in extracellular degradation and clearance may contribute to lower circulating MPO-DNA levels. Therefore, the present data do not establish an independent relationship between circulating neutrophil abundance and MPO-DNA levels, and these potential mechanisms require prospective validation.

The absence of correlation between Cit-H3 and MPO-DNA complexes highlights an important issue in NET-related biomarker research. Although both markers are frequently used as indicators of NET formation, they represent different molecular components and may be differentially affected by release mechanisms, nuclease activity, proteolytic degradation, serum stability, and clearance (17–19). Our findings support the view that NET-related biomarkers should be interpreted individually rather than treated as interchangeable measures of a single biological process.

These observations may have implications for understanding neutrophil-associated immune remodeling in multiple myeloma. The myeloma microenvironment is shaped by both adaptive and innate immune alterations (7, 20, 21). While most immune studies have focused on lymphoid cells, macrophages, and myeloid-derived suppressor cells, our findings suggest that neutrophil-related circulating signals may also vary across disease states. Future studies integrating serum NET-related biomarkers with bone marrow neutrophil phenotyping, spatial assessment of NET deposition, and single-cell immune profiling will be important to define the tissue-level relevance of these circulating signatures.

Interestingly, neither Cit-H3 nor MPO-DNA was significantly associated with established myeloma risk stratification systems, including R-ISS and R2-ISS, or with high-risk cytogenetic abnormalities [del(17p), t(4;14), or 1q21 amplification]. This suggests that circulating NET-related markers may reflect host immune-inflammatory or hematopoietic features rather than conventional plasma cell-intrinsic risk markers. However, these analyses were limited by the available NDMM sample size and should be validated in larger cohorts.

Exploratory survival analyses did not demonstrate statistically significant associations between baseline NET-related markers and PFS or OS. Although patients with a high MPO-DNA/low Cit-H3 profile showed numerically favorable outcomes, the subgroup size was very small and the analysis was underpowered. Therefore, these findings should be regarded as hypothesis-generating rather than prognostic evidence.

An important consideration is that circulating NET-related biomarkers in patients with multiple myeloma are likely influenced by multiple real-world factors, including disease status, marrow reserve, prior therapy, corticosteroid exposure, occult infection, comorbidities, renal function, and systemic inflammation. These factors are difficult to fully disentangle in a retrospective cohort and may interact with one another. Therefore, the present results should be interpreted as clinical serum biomarker associations rather than mechanistic readouts of NET formation. This limitation is also clinically relevant: in real-world myeloma practice, circulating NET-related biomarkers are unlikely to reflect a single biological process. The divergent patterns of Cit-H3 and MPO-DNA observed here underscore the need for cautious and marker-specific interpretation. The real-world heterogeneity of the cohort may be viewed not only as a limitation but also as a clinically relevant context for biomarker interpretation. In routine practice, serum NET-related markers are measured in patients with variable disease burden, marrow reserve, treatment exposure, and inflammatory status. The lack of concordance between Cit-H3 and MPO-DNA in this setting highlights the importance of interpreting individual NET-related biomarkers separately rather than assuming that they represent a uniform NET signal.

Several limitations should be acknowledged. First, this was a retrospective single-center study with a relatively small healthy control cohort. Second, circulating Cit-H3 and MPO-DNA complexes were measured in serum and may be influenced by sample processing, clotting-associated ex vivo events, extracellular degradation, and clearance. Therefore, these biomarkers should not be interpreted as direct measurements of tissue-level NET formation or bone marrow neutrophil activity. Third, functional neutrophil assays, spatial assessment of NET deposition, and bone marrow immune phenotyping were not performed. Fourth, because this was a real-world cohort, residual confounding related to prior therapy, corticosteroid exposure, occult infection, comorbidities, renal dysfunction, marrow reserve, and systemic inflammation could not be fully excluded. Finally, the survival analyses were limited by incomplete follow-up and small subgroup sizes. These limitations underscore the need for prospective studies integrating circulating biomarkers with mechanistic and tissue-level immune analyses.

In conclusion, circulating Cit-H3 and MPO-DNA complexes showed divergent immune-associated profiles in multiple myeloma. Cit-H3 was linked to relapsed/refractory disease and systemic immune-inflammatory imbalance, whereas MPO-DNA complexes were associated with preserved hematologic status and lower tumor burden. These findings indicate that circulating NET-related markers are not interchangeable and may capture distinct aspects of neutrophil-associated immune perturbation in multiple myeloma. Prospective studies integrating serum biomarkers, bone marrow immune profiling, and functional neutrophil assays are warranted to clarify the role of NET-related immune signatures in myeloma progression and therapeutic response.

## Data Availability

The raw data supporting the conclusions of this article will be made available by the authors, without undue reservation.
